# The Clinicians’ Approach to Documenting Functional vs. Physical Symptoms in a Tertiary Centre: A Service Evaluation.

**DOI:** 10.1192/bjo.2026.11637

**Published:** 2026-06-30

**Authors:** Amy Jaiteh, Louisa Draper

**Affiliations:** Alder Hey Children's Hospital, Liverpool, United Kingdom

## Abstract

**Aims:**

This service evaluation aimed to assess whether discrepancies exist in how functional symptoms are documented and discussed when compared to assessment of physical health symptoms.

The primary hypotheses were: (1) the documentation of functional symptoms would be less consistent than documentation of physical symptoms; (2) that functional diagnoses would be less likely to be documented as discussed with patients and their care givers than physical health symptoms; (3) physical health diagnoses were more likely to be recorded on the electronic diagnosis list than functional symptoms or diagnoses; and (4) that mental health professionals would demonstrate the most consistent approach in documentation and communication.

**Methods:**

Patients (n=4) were identified by the trust’s consultant liaison psychiatrist. Selection criteria were; (1) have a recorded diagnosis of Functional Neurological Disorder or recorded functional symptoms and (2) have accessed care from a department at the tertiary centre in the last 12 months.

A systematic retrospective review of each patient’s electronic clinical record was conducted, examining all documented clinical encounters. Documentation was analysed across clinical letters, clinical notes, referrals, and the electronic problem list.

From each clinical document, the diagnoses recorded, the diagnostic language used, and the documented discussions were recorded. Tabulation allowed for comparison or quantitative data and summary narratives were composed to enable thematic analysis of qualitative data.

**Results:**

The evaluation demonstrated marked inconsistency in the documentation of functional symptoms compared to physical symptoms supporting the primary hypotheses. Differences also existed between clinician groups and the proportion of diagnosis discussions documented.

Functional diagnoses were less likely to be explicitly named, discussed with families, or recorded on the formal diagnosis list compared with physical diagnoses. Clinicians frequently used descriptive or tentative language when documenting functional symptoms. In contrast, physical symptoms were documented consistently, with clear diagnostic labels and subsequent discussions with patients and families. Mental health professionals showed greater consistency in documenting functional diagnoses than physical health specialists.

**Conclusion:**

This service evaluation supports the hypothesis that functional symptoms are managed and documented less consistently than physical symptoms within tertiary paediatric care. The findings highlight a clear need for a standardised approach to the documentation of and discussion regarding all diagnosed medical conditions. Differences in the documentation approach from physical health and mental health specialists can be mediated by targeted local training and revisions to the electronic documentation systems. Establishing a dedicated functional service led by a specialist multi-disciplinary team may improve diagnostic confidence, documentation consistency, and patient experience.

